# LONG TERM RESULTS AFTER STAPLED HEMORRHOIDOPEXY ALONE AND COMPLEMENTED BY EXCISIONAL HEMORRHOIDECTOMY: A RETROSPECTIVE COHORT STUDY

**DOI:** 10.1590/0102-6720201600030008

**Published:** 2016

**Authors:** Sergio Eduardo Alonso ARAUJO, Lucas de Araujo HORCEL, Victor Edmond SEID, Alexandre Bruno BERTONCINI, Sidney KLAJNER

**Affiliations:** Hospital Israelita Albert Einstein, São Paulo, SP, Brazil

**Keywords:** Hemorrhoids, Hemorrhoidectomy, Rectal prolapse, Stapled hemorrhoidopexy, Recurrence

## Abstract

**Background:**

Stapled hemorrhoidopexy is associated with less postoperative pain and faster recovery. However, it may be associated with a greater risk of symptomatic recurrence. We hypothesized that undertaking a limited surgical excision of hemorrhoid disease after stapling may be a valid approach for selected patients.

**Aim:**

To compare long-term results after stapled hemorrhoidopexy with and without complementation with closed excisional technique.

**Method:**

In a retrospective uni-institutional cohort study, sixty-five (29 men) patients underwent stapled hemorrhoidopexy and 21 (13 men) underwent stapled hemorrhoidopexy with excision. The same surgeons operated on all cases. Patients underwent stapled hemorrhoidectomy associated with excisional surgery if symptoms attributable to external hemorrhoid piles were observed preoperatively, or if residual prolapse or bulky external disease was observed after the firing of the stapler. A closed excisional diathermy hemorrhoidectomy without vascular ligation was utilized in all complemented cases. All clinical variables were obtained from a questionnaire evaluation obtained through e-mail, telephone interview, or office follow-up.

**Results:**

The median duration of postoperative follow-up was 48.5 (6-40) months. Patients with grades 3 and 4 hemorrhoid disease were operated on more frequently using stapled hemorrhoidopexy complemented with excisional technique (95.2% vs. 55.4%, p=0.001). Regarding respectively stapled hemorrhoidopexy and stapled hemorrhoidopexy complemented with excision, there was no difference between the techniques in relation to symptom recurrence (43% and 33%, p=0.45) and median interval between surgery and symptom recurrence (30 (8-84) and 38.8 (8-65) months, p=0.80). Eight (12.3%) patients were re-operated after stapled hemorrhoidopexy and 2 (9.6%), after hemorrhoidopexy with excision (p=0.78). Patient distribution in both groups according to the degree of postoperative satisfaction was similar (p=0.97).

**Conclusion:**

Stapled hemorrhoidopexy combined with an excisional technique was effective for more advanced hemorrhoid disease. The combination may have prevented symptomatic recurrence associated to stapled hemorrhoidopexy alone.

## RESUMO

**Racional:**

A hemorroidopexia com grampeador está significativamente associada a menor dor pós-operatória e recuperação mais rápida. No entanto, há evidência de que possa ter maior taxa de recorrência sintomática. A hipótese é que empreender excisão cirúrgica limitada da doença hemorroidária após o grampeamento pode ser abordagem válida para pacientes selecionados.

**Objetivo:**

Avaliar os resultados comparativos a longo prazo após a técnica de grampeamento isolada ou associada à complementação por técnica excisional fechada.

**Método:**

Coorte retrospectiva, com 65 (29 homens) pacientes submetidos à hemorroidopexia por grampeamento e 21 (13 homens) a ela complementada por técnica excisional. Os pacientes foram submetidos à complementação do grampeamento se sintomas atribuíveis às hemorróidas externas fossem observados no pré-operatório, ou se prolapso residual ou doença externa volumosa existisse após o disparo do grampeador. A hemorroidectomia excisional fechada por eletrocauterização sem ligadura vascular foi utilizada em todos os casos de complementação. Todas as variáveis ​​clínicas foram obtidos a partir de um questionário eletrônico, entrevista por telefone, ou em consulta.

**Resultados:**

A duração média do seguimento pós-operatório foi de 48,5 (6-40) meses. Os pacientes com graus hemorroidários 3 e 4 foram operados com mais frequência usando grampeamento complementado por técnica excisional (95,2% vs. 55,4% - p=0,001). Os pacientes operados por grampeamento sem e com complementação por técnica excisional, não houve diferença entre os grupos quanto a recidiva dos sintomas (43% vs. 33%, p=0,45, respectivamente) ou quanto ao intervalo médio entre a opração e a recorrência dos sintomas (30 (8-84) vs. 38,8 (8-65) meses, p=0,80). Oito (12,3%) pacientes foram reoperados após grampeamento isolado e 2 (9,6%), após grampeamento complementado por técnica excisional (p=0,78). O grau de satisfação pós-operatória foi semelhante entre as técnicas (p=0,97).

**Conclusão:**

A hemorroidopexia por grampeamento combinada com técnica excisional foi eficaz para a doença hemorróida mais avançada. A combinação pode ter impedido a recorrência sintomática associada a hemorroidopexia isolada.

## INTRODUCTION

Conventional excisional hemorrhoidectomy has proven to be very effective as a long-term alternative for hemorrhoid disease therapy. The Milligan-Morgan operation is currently the standard approach for hemorrhoid prolapse in Europe, while the Ferguson closed hemorrhoidectomy is the operation of choice in North America. Although highly effective for long-lasting symptomatic control, excisional hemorrhoidectomy is associated with significant postoperative pain, which remains the most important postoperative complication and the leading cause for deferral of treatment.

Stapled hemorrhoidopexy (SH) was introduced in 1998^18^ as an alternative to excisional hemorrhoidectomy techniques. It has revolutionized the traditional surgical approach to hemorrhoid disease by introducing the concept of dealing with the rectal mucosal prolapse by resecting a mucosal cylinder above the dentate line through mechanical stapling^13^. It represents a non-excisional approach for the surgical treatment of hemorrhoid disease. In this procedure, it is aimed at repositioning the prolapsed hemorrhoid tissue through a circular resection of the inner layers (mucosa, submucosa, and part of the muscularis propria). In association, the mechanical anopexy would also cause an interruption of the vascular supply to the hemorrhoid cushions leading to a volume reduction of the hemorrhoid tissue. 

SH was studied in several randomized controlled trials^3^ ^,^ ^4^ ^,^ ^15^ ^,^ ^21^ ^,^ ^24^ ^,^ ^28^ in which its safety and early-term efficacy has been demonstrated. Systematic reviews of randomized controlled trials followed by meta-analyses have demonstrated that the short-term outcomes results favor SH when compared to traditional excisional techniques^4^ ^,^ ^5^ ^,^ ^14^ ^,^ ^19^ ^,^ ^22^ ^,^ ^27^. Chiefly, SH is associated to shorter operative time, reduced inpatient stay, less pain, and earlier return to normal activities^25^. However, meta-analyses of randomized controlled trials have evinced that SH may be associated with a higher symptomatic recurrence rate when compared to conventional excisional techniques^6^ ^,^ ^1^ ^,0,^ ^17^ ^,^ ^25^ ^,^ ^27^. These results led Giordano et al.^10^ to conclude that patients should choose whether to accept a higher risk of recurrence and additional operation for the sake of the short-term benefits of SH compared with conventional hemorrhoidectomy.

There are important issues to be considered when reviewing the studies included in the systematic reviews concluding for higher recurrence associated with SH when compared to conventional hemorrhoidectomy. One derives from the heterogeneity in the diagnosis of hemorrhoid disease grade^16^. The second is that it must be noted that many of the randomized trials included in these reviews recruited very few patients^4^ ^,^ ^7^ ^,^ ^12^ ^,^ ^15^ ^,^ ^21^. Therefore, it seems reasonable to assume that a comparison of apples and oranges was undertaken in these trials in the following sense: patients included in the SH group were most certainly the very first ones operated on by the participant surgeons using stapling devices. In other words, in these studies, the previous clinical experience (learning curve) with SH was not declared. On the other hand, participating surgeons entering these trials have most likely reached expert level in conventional techniques. Ultimately, randomized assortment is incapable of solving the effect of different learning curves.

In spite of this controversy, SH has been successfully used for the surgical management of hemorrhoids since 1999 in our institution. However, surgical indications for SH may not be the same of conventional excisional techniques. Therefore, was hypothesized that undertaking a limited surgical excision of hemorrhoid disease after stapling in the same surgical procedure may be a valid approach for selected patients. The potential advantages of the combined technique are the technical simplicity for the management of internal hemorrhoids through stapling, and the reduced risk of symptomatic recurrence due to excision of external hemorrhoids. 

The aim of this study was to compare long-term results after stapled hemorrhoidopexy with (SH+E) and without (SH) complementation with closed excisional technique.

## METHODS

The study was reviewed and approved by the Institutional Review Board at Hospital Israelita Albert Einstein, Sao Paulo, SP, Brazil. It represents a sole institutional retrospective cohort study. The study included consecutive patients who underwent stapled SH or SH+E operated on from January, 2011 through December, 2014. All study participants provided written informed consent prior to study enrollment.

The primary endpoint of this study was symptomatic recurrence. Recurrence was characterized according to the following variables: 1) time interval between symptomatic recurrence and questionnaire evaluation; 2) presence of any symptoms related to hemorrhoid disease in the month previous to questionnaire evaluation; and 3) need for medical treatment or reoperation during the follow-up period. 

The second endpoint was degree of satisfaction with surgical treatment of hemorrhoid disease.

Eligibility criteria for participating in this unmatched cohort study were: patients of either gender who had undergone surgical treatment of hemorrhoids through SH or SH+E evaluated through a standardized questionnaire assessment, and being mentally capable of understanding the questions. Patients were excluded if the standardized clinical questionnaire could not be fulfilled, if associated anal surgery had been undertaken at the time of surgical treatment of hemorrhoids, or if the presence of other anal condition was suspected or diagnosed at the time of questionnaire evaluation.

The indication for SH was non-fixed circumferential hemorrhoid prolapse. Patients underwent SH+E if symptoms attributable to external hemorrhoid piles were observed preoperatively or if bulky external disease was observed right after firing the stapler. Symptoms attributed to external hemorrhoid disease were pain, itching, and episodes of external hemorrhoid thrombosis. 

All clinical variables were collected from a standardized questionnaire evaluation obtained through e-mail, telephone interview or office follow-up conducted in all cases by the same author (LAH). The following variables were recorded in all cases: age, gender, grade of hemorrhoid disease, previous treatment, type of surgical treatment (SH or SH+E), duration of follow-up (time interval in months between surgery and questionnaire evaluation), time interval in months between surgery and symptomatic recurrence, presence of symptoms in the month before questionnaire evaluation, need for and frequency of medical treatment in the postoperative period, degree of satisfaction, and need for reoperation. 

### Surgical procedures

No bowel preparation was used. Antibiotic prophylaxis was used in all patients. All operations were performed under spinal or general anesthesia. Patients were operated on in the lithotomy position. The same surgeon (SEAA) has operated on all patients.

SH was performed as described in the literature^1^. A 2/0 polypropylene pursestring suture including the mucosa and submucosa was applied 2 cm above the dentate line. Mucosectomy and anopexy was conducted using the PPH-03 kit (Ethicon Endo-Surgery, Cincinnati, OH, USA) with closed staple height of 0.75 mm (rather than 1 mm in PPH-01) in all cases. Once the pursestring suture is in place, the circular stapler is introduced to the anus. The stapler is opened to its maximum position, and the head positioned proximal to the suture. The suture is tied with a closing knot and the ends are pulled through the lateral holes of the stapler. It is knotted externally or fixed using a clamp, and tightened onto the shaft. The entire casing of the stapler is introduced into the anal canal, and moderate traction put on the pursestring to draw the prolapsed mucous membrane into the casing of the stapler. The instrument is then tightened and fired. 

The SH+E procedure is a combined operation. After a complete SH, an "economic" closed excisional hemorrhoidectomy is undertaken using electrocautery dissection with no vascular pedicle ligation as previously described^21^. Excision is performed with the hemorrhoid in its anatomical position, and the wound is closed using a continuous 4/0 polyglactin suture. It is important to emphasize that in this group, the complementary closed hemorrhoidectomy does not represent excision of skin tags. 

Patients were routinely discharged in the day after the operation.

### Statistical analysis

A biomedical statistician conducted the statistical review of the present study. The Fisher's exact test was used for comparison between SH and SH+E groups regarding: patient gender, grade of hemorrhoid disease (1 to 4), previous treatment (none, rubber band ligation, or hemorrhoidectomy), presence of symptoms in the month before questionnaire evaluation (yes, no), need for and frequency of demanded medical treatment in the postoperative period (no, rarely, occasionally, frequently, daily), and indication/type of reoperation (none, hemorrhoidectomy, resection of anal tags, and treatment of anal stenosis). The F-test of equality of variances was used for comparison between SH and SH+E groups regarding patient age, duration of follow-up (interval between surgery and questionnaire assessment), and time until symptomatic recurrence. Results were expressed as median (interval) for continuous variables. Statistical testing was undertaken considering p values <0.05 to be significant.

## RESULTS

### Patient characteristics

Sixty-five (29 men) patients underwent SH, and 21 (13 men) SH+E - p=0.22. In the SH group, mean age was 50 (range, 22-83); in the SH+E, 48.3 (range, 23-67) - p=0.66.

Regarding hemorrhoid disease grade, the distribution of disease grades 1 to 4 was significantly different between the two treatment groups (Table 1). Grades 3 and 4 were most frequently observed among patients undergoing SH+E - p=0.002.

**TABLE 1:** Distribution of hemorrhoid disease grade according to treatment group

Grade	SH group		SH+E group		Total		p
	n	(%)	n	(%)	n	(%)	
1	4	6.2	0	0	4	4.7	
2	25	38.5	1	4.8	26	30.2	
3	30	46.2	13	61.9	43	50	
4	6	9.2	7	33.3	13	15.1	
Total	65	100	21	100	86	100	0.002

SH=stapled hemorrhoidopexy; SH+E=stapled hemorroidopexy with excision

The mean postoperative follow-up duration in the present study was 48.5 (range, 6-40) months. No difference was observed between the mean follow-up duration after SH (37.1 months, range 6-42), and after SH+E (39 months, range 6-40) - p=0.79.

Of all 86 patients, 68 (79%) have not undergone previous treatment before SH or SH+E. For patients undergoing SH nine (13.8%) underwent rubber band ligations; four (6.2%) surgical hemorrhoidectomy, and 52 (80%), no intervention. In the SH+E group, these numbers were, respectively, 3 (14.3%), 2 (9.5%), and 16 (76.2%), p=0.80.

### Primary endpoint-related variables

**FIGURE 1:**
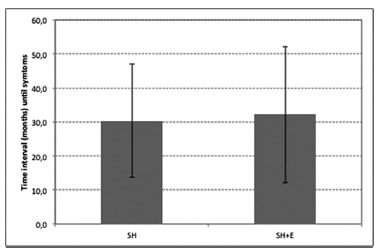
Mean time interval (months) between surgical treatment and symptomatic recurrence (p=0.80)

Regarding the mean time interval between surgery for hemorrhoids and symptomatic recurrence, no significant difference (p=0.80) was observed between SH (30.3 months; range, 8-84) and SH+E groups (32.1 months; range, 8-65, Figure 1).

**FIGURE 2:**
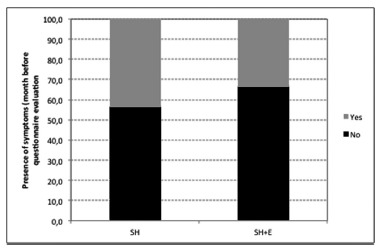
Presence of symptoms in the month before questionnaire evaluation (p=0.46)

In the SH group, 28 (43.1%) patients have declared presence of symptoms potentially related to hemorrhoid disease during the month preceding questionnaire assessment. In the SH+E group, seven (33.3%) patients have answered the same way, p=0.46 (Figure 2).

**FIGURE 3:**
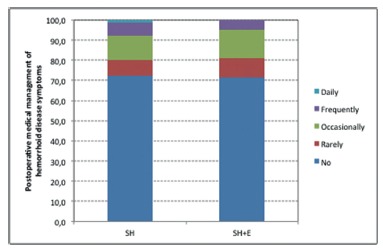
Need for and frequency of medical treatment in the postoperative follow-up period (p=0.99)

Seventy-two percent (62 of 86 patients) of all operated patients in the present study reported no need for medical treatment after a median follow-up of 38.5 (6-40) months. In the SH group, of 29 patients, medical management of symptoms related to hemorrhoid disease in the postoperative period was rarely required in 5 (7.7%), occasionally, in 8 (12.3%); frequently, in 4 (6.2%); and daily, in 1 (1.5%). In the SH+E group, of 21 patients, these numbers were, respectively, 2 (9.5%), 3 (14.3%), 1 (4.8%), and 0 - p=0.99 (Figure 3).

**FIGURE 4:**
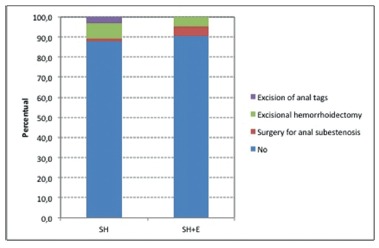
Type of reoperation within the follow-up period (p=0.78)

Eighty-eight percent (76 of 86 patients) of surgically managed patients in this study were not re-operated until the end of the follow-up period. In the SH group, of 29 patients, 1 (1.5%) underwent surgery for anal subestenosis, 5 (7.7%) for excisional hemorrhoidectomy, and 3 (3.1%) had resection of anal tags. In the SH+E, of 21 patients, these results, were respectively, 1 (4.8%), 1 (4.8%), and 0, p= 0.78 (Figure 4).

### Secondary endpoint results: degree of satisfaction

**FIGURE 5:**
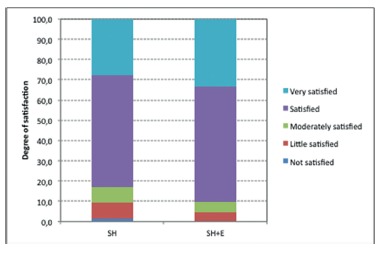
Degree of satisfaction with the surgical treatment of hemorrhoids (p=0.97)

Of 86 patients included and followed in the present study, 25 (29.1%) stated that they were "very satisfied" with the outcome; 48 (55.8%) that they were "satisfied" with the result of their surgery; 6 (7%) declared themselves "moderately satisfied"; another 6 (7%) patients, "little satisfied", and 1 (1.2%) was "not satisfied". No difference between groups SH and SH+E, regarding late degree of satisfaction, p=0.97 was observed (Figure 5). 

## DISCUSSION

In this retrospective cohort study, was observed that stapled hemorrhoidopexy combined with an excisional technique was effective for more advanced hemorrhoid disease and might have prevented symptomatic recurrence for this subset of patients with grades 3 and 4 disease. 

The ideal treatment for hemorrhoids should be minimally invasive, painless, safe and effective. There remains an extensive discussion regarding stapled hemorrhoidopexy and, most recently, other forms of non-excisional hemorrhoid surgery, such as the Doppler-guided transanal hemorrhoid dearterialization^13^ especially regarding late recurrence rates after surgery.

In an appropriate systematic review and economic evaluation of SH, the technique was associated with less pain in the immediate postoperative period, but with a higher rate of residual prolapse, prolapse in the longer term and prolapse re-intervention^5.^. Moreover, patients affected by third degree hemorrhoids were ten times more likely to develop recurrences, and, in general, twice as likely to undergo further treatment to correct recurrent prolapses^2^ ^,^ ^15^. As result, Nisar et al.^19^ declared that conventional hemorrhoid surgery remained the gold-standard for the surgical management of hemorrhoids. Ultimately, Giordano et al. have stated that it's a patient choice whether to accept a higher late recurrence rate to take advantage of the short-term benefits of SH^10^.

Perhaps, the stated above is not all that has been left for the patient willing to undergo surgery based on a non-excisional procedure for the cure of hemorrhoids. In the present retrospective cohort study was demonstrated for the first time that a combination of SH and excisional hemorrhoidectomy in the same patient using strict selected criteria may be associated with a symptomatic recurrence rate similar to that observed after SH alone. This finding may represent evidence favoring the perception that not all cases of hemorrhoids may respond well to isolated SH. In this study, was confirmed the hypothesis that when symptoms attributable to external hemorrhoid disease are observed preoperatively, or if bulky external disease is found after firing the stapler, the association of SH+E may represent a good preventive measure to avoid recurrence associated with isolated SH.

The available publications regarding the effectiveness of SH largely deal with short-term follow-up. In the available studies, follow-up periods range from six months to two years. Only few studies have reported longer follow-up periods^2^ ^,^ ^8^ ^,^ ^11^ ^,^ ^23^ ^,^ ^26^ ^,^ ^28^. With the exception of the recent publication of Kim et al.^16^, SH seems associated with a higher rate of residual symptoms and symptomatic recurrence when compared with excisional techniques^2^ ^,^ ^8^ ^,^ ^23^ ^,^ ^28^. Mixed-case population, comparison of different excisional techniques and also technical problems possibly due to a short learning curve of participating surgeons may play an important role regarding the long-term outcome of SH. Against this background, the median follow-up period in this study (48.5 months) represents a meaningful contribution to the literature although it may not be the study of SH with longer follow-up available^20^.

SH represents a relatively simple and fast operation, especially when compared to the transanal dearterialization procedure^13^. However, due to existing evidence, one cannot rule out that technical errors may play a role in the higher symptomatic recurrence rate when compared to excisional hemorrhoidectomy. Regarding technical aspects of the operation, there is significant difficulty in estimating the amount of mucosal prolapse to be removed. Moreover, it is reasonable to assume that a higher degree of hemorrhoid prolapse requires a larger resection of rectal mucosa. As result, there is increasing consensus about the concept that fourth degree hemorrhoid disease should not be a valid indication of SH. In the present study, for patients undergoing SH+E, there was a significant higher proportion of patients with hemorrhoid disease grades 3 and 4 as compared to the group of patients undergoing SH. It is believed that choosing the combined procedure (SH+E) in these cases may have prevented symptomatic recurrence.

This study has limitations. The small sample reflects the difficulty at obtaining long-term follow-up results after surgery for hemorrhoids in our midst. In addition, the retrospective nature of this study may undermine the formation of comparable groups regarding features such as preoperative office treatment, degree of hemorrhoid disease, and type of surgery. Nevertheless, in this series, the analysis of office treatments offered to patients indicated no difference between the two groups. Regarding the different distribution of the degree of hemorrhoid disease in the two groups, this result was expected. It probably represents the main reason why two distinct cohorts could be constituted and analyzed. A final consideration must be addressed regarding immediate results of SH+E, which have not been addressed in the present study, and in no other as far as is known. The authors agree that immediate results of SH+E deserve further detailed description in another paper. However, it turns out that the primary endpoint of the present study was long-term outcome results and, although it was not reported, short-term results were mostly uneventful for both groups.

In the present study, the diagnosis of recurrence derived from a set of clinical variables results, as defined previously by others^16^ ^,^ ^20^. However, the reoperation rate for residual prolapse remains an objective and credible indicator of long-term efficacy of hemorrhoid surgery. Systematic reviews have shown that the reoperation rate is higher after SH than after excisional hemorrhoidectomy^6^ ^,^ ^17^ ^,^ ^25^. Due to the controversy regarding differentiating between surgical re-treatments due to recurrent prolapse or anal skin tags^9^, was found useful to properly identify these patients. Although excisional hemorrhoidectomy was accomplished in five patients in the SH group, and in one in the SH+E, there was no difference between the two groups. In this series, excisional hemorrhoidectomy was deemed necessary when a properly diagnosed (clinical complaint and anoscopy results) recurrent prolapse was identified. As a mater of fact, was found no difficulty to designate a surgical re-intervention to these patients. Even when facing newly development changes in the regular follow-up of hemorrhoids surgery, it is believed that the comparison of the pure reoperation rate between the two groups is worthwhile. 

To our knowledge, this is the first study comparing late outcomes of patients undergoing SH in comparison with SH+E. We believe that the indications for combining SH with an economic excisional hemorrhoidectomy in our practice were well described. This combination technique has been used selectively since the awareness of early evidences regarding a superior recurrence rate associated with SH. We consider that choosing between an operation associated with better immediate outcomes but with a higher risk of recurrent symptomatic prolapse should not be the patient's choice. Therefore, it was demonstrated that combining SH and closed excisional technique places side by side a highly efficient procedure for prolapse (SH) and a largely known effective operation (excisional procedure) for external hemorrhoids. Ultimately, this option may be accessible to many patients and surgeons.

## CONCLUSION

Stapled hemorrhoidopexy combined with an excisional technique was effective for more advanced hemorrhoid disease. The combination may have prevented symptomatic recurrence associated to SH alone. 
